# Risks and Benefits of Feeding Enterostomy Creation During Minimally Invasive Esophagectomy: A Propensity‐Weighted Analysis Using the Japanese National Clinical Database

**DOI:** 10.1002/ags3.70216

**Published:** 2026-03-17

**Authors:** Eisuke Booka, Shinya Hirakawa, Hisateru Tachimori, Koji Tanaka, Hideki Ueno, Yasue Kimura, Ken Shirabe, Hiroya Takeuchi

**Affiliations:** ^1^ Department of Surgery Hamamatsu University School of Medicine Shizuoka Japan; ^2^ Department of Health Policy and Management Keio University School of Medicine Tokyo Japan; ^3^ Department of Healthcare Quality Assessment, Graduate School of Medicine The University of Tokyo Tokyo Japan; ^4^ Project Management Subcommittee The Japanese Society of Gastroenterological Surgery Tokyo Japan; ^5^ Department of Surgery, Gastroenterological Surgery, Graduate School of Medicine Osaka University Osaka Japan; ^6^ Database Committee The Japanese Society of Gastroenterological Surgery Tokyo Japan; ^7^ Department of Surgery National Defense Medical College Saitama Japan; ^8^ The Japan Esophageal Society Tokyo Japan; ^9^ Department of Gastroenterological Surgery National Hospital Organization Kyushu Cancer Center Fukuoka Japan; ^10^ The Japanese Society of Gastroenterological Surgery Tokyo Japan; ^11^ Division of Hepatobiliary and Pancreatic Surgery, Department of General Surgical Science, Graduate School of Medicine Gunma University Maebashi Japan

**Keywords:** esophageal cancer, feeding enterostomy, gastrostomy, jejunostomy, minimally invasive esophagectomy

## Abstract

**Background:**

Feeding enterostomy is commonly created during minimally invasive esophagectomy (MIE); however, its short‐term impact remains unclear.

**Methods:**

We analyzed 19 054 patients who underwent MIE for esophageal or esophagogastric junction cancer during 2019–2022. Inverse probability of treatment weighting was applied to balance baseline characteristics, and G‐computation was used to estimate adjusted risks and means and their differences. A secondary analysis was performed to compare gastrostomy and jejunostomy in retrosternal cases.

**Results:**

Of 19 054 patients, 4599 (24.1%) received a feeding enterostomy. After adjustment, the primary outcome, postoperative bowel obstruction, did not differ significantly between enterostomy group and no‐enterostomy group (+0.2%, *p* = 0.132). The enterostomy group demonstrated higher rates of reoperation (+2.5%, *p* < 0.001) and respiratory complications, including pneumonia (+2.5%, *p* < 0.001) and prolonged ventilation (+0.9%, *p* = 0.012), than the no‐enterostomy group. Conversely, delayed gastric emptying (−0.9%, *p* < 0.001) and deep vein thrombosis (−0.4%, *p* = 0.028) occurred less frequently. Among 2723 patients who underwent retrosternal reconstruction with feeding enterostomy, jejunostomy was associated with a shorter operative time (−11.2 min, *p* = 0.025), whereas gastrostomy was associated with a 2.3‐day shorter hospital stay than jejunostomy (*p* = 0.022). Bowel‐related events were rare, and adjusted comparisons for these outcomes were not performed.

**Conclusion:**

Feeding enterostomy during MIE may confer benefits (e.g., reduced delayed gastric emptying and deep vein thrombosis) but is also associated with increased postoperative complications. Routine or uniform placement of a feeding enterostomy should be avoided, and gastrostomy may be preferable in retrosternal reconstruction.

## Graphical abstract

Feeding enterostomy during MIE was evaluated in 19 054 patients from the Japanese NCD using propensity weighting. Enterostomy was associated with higher reoperation and respiratory complications, but lower delayed gastric emptying and deep vein thrombosis, with no significant difference in overall bowel obstruction. In retrosternal reconstruction, gastrostomy was associated with shorter hospital stay and tended to have fewer bowel‐related events, suggesting that routine enterostomy placement should be avoided and enteral access should be individualized.

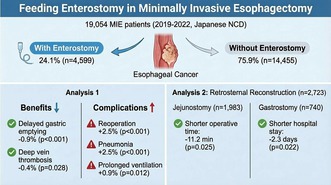

## 1 Introduction

Esophagectomy for esophageal cancer is a highly invasive procedure associated with substantial postoperative morbidity and nutritional challenges [1]. In the era of open esophagectomy, prophylactic feeding enterostomy (via gastrostomy or jejunostomy) was commonly performed to enable early enteral nutrition and mitigate perioperative complications [2, 3]. This approach was widely supported in the literature and continues to be used in patients at high risk of postoperative dysphagia, insufficient oral intake, or delayed recovery [4, 5, 6, 7].

With advances in surgical techniques, minimally invasive esophagectomy (MIE), including thoracoscopic and robot‐assisted approaches, has become the standard procedure in many institutions due to reduced surgical trauma and improved recovery profiles [8]. Concurrently, perioperative care has evolved with the implementation of enhanced recovery protocols and early oral intake strategies [9]. These developments have prompted an ongoing debate regarding the routine necessity of feeding enterostomy during MIE.

Although feeding enterostomy continues to be selectively used in high‐risk patients, the procedure itself carries potential complications, including infection, leakage, and postoperative bowel obstruction. The choice of enterostomy type is influenced by the reconstruction route: jejunostomy is almost exclusively used in posterior mediastinal reconstruction, whereas both gastrostomy and jejunostomy are feasible options in retrosternal reconstruction [10, 11]. Therefore, comparisons between enterostomy types should be limited to patients undergoing retrosternal reconstruction, in which both techniques are feasible.

Herein, we evaluated the short‐term outcomes associated with feeding enterostomy creation in patients undergoing MIE, using a large‐scale national clinical database (NCD). The primary analysis involved a comparison of postoperative outcomes between patients with and without feeding enterostomy, whereas a secondary analysis focused on patients undergoing retrosternal reconstruction and compared outcomes between gastrostomy and jejunostomy. Although enterostomy may provide nutritional benefits, this study aimed to clarify its balance of risks and benefits in contemporary MIE practice.

## 2 Methods

### 2.1 Data Source and Study Population

We conducted a retrospective cohort study using data from the Japanese NCD, which captures detailed clinical and surgical information for > 95% of esophagectomy cases nationwide. Patients who underwent esophagectomy with reconstruction (predominantly gastric conduit) for esophageal or esophagogastric junction cancer between January 2019 and December 2022 were analyzed.

To reflect contemporary surgical practice, the analysis was restricted to patients who underwent MIE, including thoracoscopic or robot‐assisted approaches, whereas those who underwent open thoracotomy were excluded [12, 13]. Patients who received both gastrostomy and jejunostomy, those with incomplete data on key variables, and those with duplicate records were excluded.

The Institutional Review Board of Hamamatsu University School of Medicine reviewed and approved the study protocol, and the requirement for individual written informed consent was waived (ID: 23–254).

### 2.2 Exposure and Study Groups

The primary exposure was feeding enterostomy creation, defined as gastrostomy or jejunostomy performed at the time of esophagectomy. Patients were categorized into enterostomy and no‐enterostomy groups (Analysis 1).

In a secondary subgroup analysis (Analysis 2), outcomes were compared between patients who received gastrostomy and those who received jejunostomy, restricted to individuals who underwent retrosternal reconstruction to minimize confounding related to the reconstruction route. Because gastrostomy cases were extremely limited in the posterior mediastinal route, resulting in substantial imbalance, comparisons in the overall cohort and within the posterior mediastinal subgroup were not performed.

### 2.3 Endpoints

The primary endpoint was postoperative bowel obstruction. Secondary endpoints included other short‐term postoperative outcomes: 30‐day mortality, operative mortality, reoperation within 30 days, pulmonary complications (including pneumonia, atelectasis, and empyema), delayed gastric emptying, ventilator support ≥ 48 h, surgical site infection (SSI), and postoperative length of hospital stay.

### 2.4 Statistical Analysis

Baseline patient characteristics and intraoperative variables were compared using Fisher's exact test for categorical variables and Wilcoxon rank‐sum test for continuous variables. Analyses were performed after excluding missing values for variables with incomplete data.

To adjust for baseline differences between the enterostomy and no‐enterostomy groups, inverse probability of treatment weighting (IPTW) based on propensity scores was applied. Propensity scores were estimated using logistic regression. Stabilized weights were used to reduce variance, and covariate balance was assessed using standardized mean differences, with absolute values < 0.1 considered indicative of adequate balance.

For both the primary analysis (Analysis 1) and the retrosternal subgroup analysis (Analysis 2), treatment effects were estimated under an average treatment effect framework. Binary outcomes were summarized as risk differences (RDs) with 95% confidence intervals (CIs) and continuous outcomes as mean differences with 95% CIs. Estimates were obtained using G‐computation with the glm_weightit and lm_weightit functions from the WeightIt package. Adjusted risks and means were calculated using the avg_predictions function in the marginaleffects package, and risk and mean differences were calculated using the avg_comparisons function. Standard errors were estimated using M‐estimation.

Subgroup comparisons between gastrostomy and jejunostomy were performed using IPTW and G‐computation exclusively within the retrosternal subgroup. Formal statistical comparisons in the overall enterostomy cohort and within the posterior mediastinal subgroup were not performed because of severe imbalance and the small number of gastrostomy cases.

All analyses were performed using R (version 4.3.3 or later). All statistical tests were two‐sided, and *p* < 0.05 was considered statistically significant.

## 3 Results

### 3.1 Patient Selection and Baseline Characteristics

**FIGURE 1:**
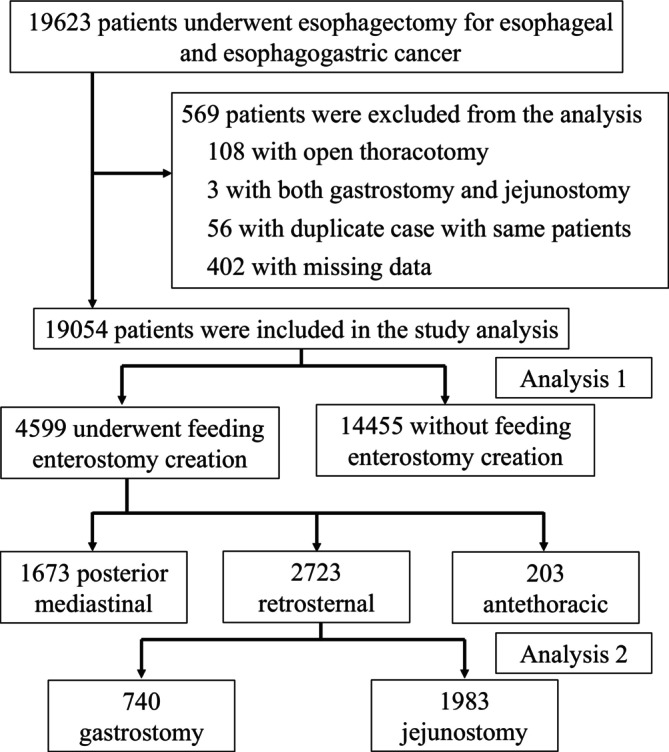
Flowchart of patient enrolment. Among the patients who participated in the Japanese National Clinical Database (NCD) between 2019 and 2022, 19 054 minimally invasive esophagectomy (MIE) cases were included in this study.

Among 3 049 466 surgical cases registered in the NCD between 2019 and 2022, 19 054 patients who underwent MIE (thoracoscopic or robot‐assisted) for esophageal or esophagogastric junction cancer were included in the final analysis after applying the exclusion criteria (Figure 1). Of these, 4599 patients (24.1%) received a feeding enterostomy (gastrostomy or jejunostomy), whereas 14 455 (75.9%) did not.

In unadjusted analyses, patients in the enterostomy group were more likely to be male, have hypertension and chronic obstructive pulmonary disease (COPD), and undergo jejunostomy with posterior mediastinal reconstruction. Surgical and anatomical characteristics, including reconstruction route and tumor location, also differed significantly between the two groups.

### 3.2 Propensity Score Weighting (IPTW) and Covariate Balance

Covariate balance was assessed for variables that could influence the decision to create a feeding enterostomy, including age, American Society of Anesthesiologists physical status (ASA‐PS), laparoscopy use, reconstruction route, and institutional volume. After IPTW, absolute standardized mean differences for all covariates were < 0.1, indicating adequate balance between the enterostomy and no‐enterostomy groups. Unweighted baseline characteristics are summarized in Table 1, and IPTW‐adjusted outcome estimates derived using G‐computation are presented in Table 2.

**TABLE 1:** Backgrounds of patients with or without feeding enterostomy.

Variables	All (*n* = 19 054) (%)	Feeding enterostomy (+) (*n* = 4599) (%)	Feeding enterostomy (−) (*n* = 14 455) (%)	*P‐value*
Male (%)	15 279 (80.2)	3739 (81.3)	11 540 (79.8)	0.030
Age (%)				0.767
< 75 y	14 466 (75.9)	3484 (75.8)	10 982 (76.0)	
≧ 75 y	4588 (24.1)	1115 (24.2)	3473 (24.0)	
BMI ≧ 25 kg/m^2^ (%)	2714 (14.2)	680 (14.8)	2034 (14.1)	0.226
Weight loss ≧ 10% (%)	1122 (5.9)	279 (6.1)	843 (5.8)	0.565
ASA‐PS grade ≧ 3 (%)	1986 (10.4)	489 (10.6)	1497 (10.4)	0.599
Smoking within 1 y (%)	6637 (34.8)	1603 (34.9)	5034 (34.8)	0.972
Habitual alcohol use (%)	13 086 (68.7)	3214 (69.9)	9872 (68.3)	0.043
COPD (%)	1447 (7.6)	540 (11.7)	907 (6.3)	< 0.001
Hypertension (%)	8352 (43.8)	2103 (45.7)	6249 (43.2)	0.003
Congestive heart failure (%)	40 (0.2)	9 (0.2)	31 (0.2)	1.000
Past cardiac surgery (%)	151 (0.8)	34 (0.7)	117 (0.8)	0.703
Cerebrovascular disease (%)	702 (3.7)	186 (4.0)	516 (3.6)	0.138
Hemodialysis (%)	53 (0.3)	11 (0.2)	42 (0.3)	0.633
Chronic steroid use (%)	215 (1.1)	48 (1.0)	167 (1.2)	0.575
Diabetes mellitus (%)				0.439
None	16 106 (84.5)	418 (9.1)	1440 (10.0)	
Diet therapy only	261 (1.4)	59 (1.3)	202 (1.4)	
Oral medication	1858 (9.8)	418 (9.1)	1440 (10.0)	
Insulin therapy	577 (3.0)	146 (3.2)	431 (3.0)	
No treatment	252 (1.3)	192 (1.3)	60 (1.3)	
Serum albumin < 2.5 g/dL (%)	114 (0.6)	18 (0.4)	96 (0.7)	0.037
Serum creatinine ≧ 1.2 mg/dL (%)	1448 (7.6)	355 (7.7)	1093 (7.6)	0.725
Tumor location				
Ce	660 (3.5)	155 (3.4)	505 (3.5)	0.711
Ut	3256 (17.1)	836 (18.2)	2420 (16.7)	0.026
Mt	9621 (50.5)	2296 (49.9)	7325 (50.7)	0.379
Lt	7573 (39.7)	1939 (42.2)	5634 (39.0)	< 0.001
Ae	2755 (14.5)	717 (15.6)	2038 (14.1)	0.013
Histology				0.246
Squamous cell carcinoma	16 308 (85.6)	3918 (85.2)	12 390 (85.7)	
Adenocarcinoma	2086 (10.9)	522 (11.4)	1564 (10.8)	
Other	484 (2.5)	104 (2.3)	380 (2.6)	
cT				0.057
T0/Tis/T1	7360 (38.6)	1789 (38.9)	5571 (38.5)	
T2	2739 (14.4)	704 (15.3)	2035 (14.1)	
T3	7592 (39.8)	1786 (38.8)	5806 (40.2)	
T4	1138 (6.0)	253 (5.5)	885 (6.1)	
cN				0.492
N0	8928 (46.9)	2131 (46.3)	6797 (47.0)	
N1	5530 (29.0)	1335 (29.0)	4195 (29.0)	
N2	3305 (17.3)	794 (17.3)	2511 (17.4)	
N3	1073 (5.6)	279 (6.1)	794 (5.5)	
Preoperative chemotherapy within 30 days (%)	3882 (20.4)	868 (18.9)	3014 (20.9)	0.004
Thoracic approach				< 0.001
Thoracoscopy	15 037 (78.9)	3833 (83.3)	11 204 (77.5)	
Robot‐assisted	4017 (21.1)	766 (16.7)	3251 (22.5)	
Abdominal approach				< 0.001
Open	4855 (25.5)	1658 (36.1)	3197 (22.1)	
Laparoscopy	12 857 (67.5)	2707 (58.9)	10 150 (70.2)	
Robot‐assisted	1342 (7.0)	234 (5.1)	1108 (7.7)	
Position at thoracoscopic surgery				< 0.001
Prone	13 431 (70.5)	3186 (69.3)	10 245 (70.9)	
Left lateral decubitus	4244 (22.3)	1215 (26.4)	3029 (21.0)	
Supine	1235 (6.5)	178 (3.9)	1057 (7.3)	
Right lateral decubitus	144 (0.8)	20 (0.4)	124 (0.9)	
Reconstruction route				< 0.001
Retrosternal	12 773 (67.0)	2723 (59.2)	10 050 (69.5)	
Posterior mediastinal	5443 (28.6)	1673 (36.4)	3770 (26.1)	
Antethoracic	838 (4.4)	203 (4.4)	635 (4.4)	
Hospital volume (annual case)				< 0.001
< 9	3464 (18.2)	761 (16.5)	2703 (18.7)	
9–18	3641 (19.1)	872 (19.0)	2769 (19.2)	
19–41	5201 (27.3)	1485 (32.3)	3716 (25.7)	
≧ 42	6748 (35.4)	1481 (32.2)	5267 (36.4)	

*Note:* Missing (unknown) categories for histology, cT, and cN were excluded from the table. Percentages were calculated using the total number of patients in each column (including missing values); therefore, category totals may not sum to 100%. P values were calculated after excluding missing values.

Abbreviations: Ae, abdominal esophagus; ASA‐PS, American Society of Anesthesiologists physical status; BMI, body mass index; COPD, chronic obstructive pulmonary disease; Ce, cervical esophagus; Lt, lower thoracic esophagus; Mt., middle thoracic esophagus; Ut, upper thoracic esophagus.

**TABLE 2:** Surgical outcomes and postoperative complications in patients with or without feeding enterostomy.

Variables	Feeding enterostomy (+) (*n* = 4599)	Feeding enterostomy (−) (*n* = 14 455)	Difference (Enterostomy − No Enterostomy)	95% CI	*P‐value*
Operation time (min)	535.0	521.9	13.1	(8.9, 17.3)	< 0.001
Blood loss (ml)	214.0	219.5	−5.6	(−15.7, 4.6)	0.282
Postoperative hospital stays (days)	28.9	28.9	−0.1	(−0.8, 0.7)	0.876
30‐day mortality (%)	0.6	0.7	−0.1	(−0.3, 0.2)	0.687
Operative mortality (%)	1.2	1.4	−0.1	(−0.5, 0.3)	0.552
Reoperation within 30 days (%)	7.0	4.5	2.5	(1.7, 3.3)	< 0.001
Bowel obstruction (%)	0.7	0.5	0.2	(−0.1, 0.5)	0.132
Reoperation due to bowel obstruction (%)	0.5	0.3	0.2	(0.0, 0.5)	0.041
Pneumonia (%)	16.7	14.2	2.5	(1.3, 3.8)	< 0.001
Atelectasis (%)	5.6	4.1	1.5	(0.7, 2.3)	< 0.001
Mechanical ventilation for more than 48 h (%)	4.8	3.9	0.9	(0.2, 1.6)	0.012
Unplanned intubation (%)	4.0	3.5	0.5	(−0.2, 1.2)	0.133
Empyema (%)	1.4	1.1	0.3	(−0.1, 0.7)	0.193
Pulmonary embolism (%)	1.0	1.0	0.0	(−0.3, 0.3)	0.931
Deep vein thrombosis (%)	1.1	1.5	−0.4	(−0.8, 0.0)	0.028
Superficial surgical site infection (%)	6.2	7.0	−0.7	(−1.6, 0.1)	0.079
Deep surgical site infection (%)	3.0	3.6	−0.5	(−1.1, 0.1)	0.090
Organ surgical site infection (%)	8.0	8.2	−0.2	(−1.2, 0.7)	0.638
Gastric necrosis	0.4	0.4	0.1	(−0.1, 0.3)	0.421
Pancreatic fistula (%)	0.4	0.4	0.0	(−0.2, 0.2)	0.824
Delayed gastric emptying (%)	2.1	3.0	−0.9	(−1.3, −0.4)	< 0.001

*Note:* Adjusted risks, means, and differences were estimated using inverse probability of treatment weighting with G‐computation.

Abbreviation: CI, confidence interval.

### 3.3 Analysis 1: Enterostomy vs. no Enterostomy

Table 1 summarizes the unweighted baseline characteristics according to the feeding enterostomy status. In the enterostomy group, thoracoscopic procedures were significantly more common in the thoracic approach, laparotomy was more frequently used in the abdominal approach, the posterior mediastinal route was more frequently used for reconstruction, and institutional volume was significantly lower than that in the no‐enterostomy group. COPD, a known risk factor for respiratory complications, was also more prevalent in the enterostomy group.

Table 2 presents the adjusted risks and RDs for major postoperative complications. Compared with the no‐enterostomy group, patients who received a feeding enterostomy had higher risks of reoperation (7.0% vs. 4.5%; RD, + 2.5%; *p* < 0.001) and respiratory complications, including pneumonia (16.7% vs. 14.2%; RD, + 2.5%; *p* < 0.001), atelectasis (5.6% vs. 4.1%; RD, + 1.5%; *p* < 0.001), and ventilator use ≥ 48 h (4.8% vs. 3.9%; RD, + 0.9%; *p* = 0.012).

Conversely, delayed gastric emptying (2.1% vs. 3.0%; RD, −0.9%; *p* < 0.001) and deep vein thrombosis (1.1% vs. 1.5%; RD, −0.4%; *p* = 0.028) were significantly less frequent in the enterostomy group. The incidence of postoperative bowel obstruction, the primary endpoint of this study, was 0.7% and 0.5% in the enterostomy and no‐enterostomy groups (RD, +0.2%; *p* = 0.132), respectively, indicating no significant difference, however reoperation due to bowel obstruction was more frequent in the enterostomy group (0.5% vs. 0.3%; RD, +0.2%; *p* = 0.041). No significant differences were observed in 30‐day mortality, operative mortality, or postoperative length of hospital stay.

### 3.4 Analysis 2: Gastrostomy vs. Jejunostomy in Retrosternal Reconstruction

Table 3 shows the distribution of feeding enterostomy type by reconstruction route. Because gastrostomy was rarely performed outside retrosternal reconstruction (> 90% of cases), IPTW in the overall cohort resulted in extreme weights and inadequate covariate balance, reflecting limited overlap between groups. Accordingly, comparisons between gastrostomy and jejunostomy were restricted to patients who underwent retrosternal reconstruction.

**TABLE 3:** Association between the reconstruction route and feeding enterostomy type.

	Gastrostomy (*n* = 789)	Jejunostomy (*n* = 3810)
Retrosternal (%)	740 (93.8)	1983 (52.0)
Posterior mediastinal (%)	32 (4.1)	1641 (43.1)
Antethoracic (%)	17 (2.2)	186 (4.9)

Among 2723 patients who received a feeding enterostomy during retrosternal reconstruction, 1983 (72.8%) underwent jejunostomy and 740 (27.2%) underwent gastrostomy. As in Analysis 1, IPTW was applied, and covariate balance assessment confirmed adequate balance between groups.

Table 4 presents surgical outcomes and postoperative complications of gastrostomy and jejunostomy in patients undergoing retrosternal reconstruction. The operative time was significantly shorter in the jejunostomy group (521.4 min vs. 532.6 min; *p* = 0.025), whereas postoperative hospital stay was significantly shorter in the gastrostomy group (26.2 days vs. 28.5 days; *p* = 0.022). Bowel obstruction and reoperation for bowel obstruction were rare, with unadjusted incidences of 0.5% vs. 0.1% and 0.4% vs. 0.1% (jejunostomy vs. gastrostomy), respectively. Owing to the small number of events, adjusted analyses for these outcomes were not conducted (data not shown). No significant differences were observed between the two groups in other postoperative complications.

**TABLE 4:** Surgical outcomes and postoperative complications of gastrostomy and jejunostomy in patients undergoing retrosternal reconstruction.

Variables	Jejunostomy (*n* = 1983)	Gastrostomy (*n* = 740)	Difference (Jejunostomy − Gastrostomy)	95% CI	*P‐*value
Operation time (min)	521.4	532.6	−11.2	(−20.9, −1.4)	0.025
Blood loss (ml)	218.9	217.2	1.7	(−22.1, 25.5)	0.890
Postoperative hospital stays (days)	28.5	26.2	2.3	(0.3, 4.2)	0.022
30‐day mortality (%)	0.7	0.8	−0.1	(−0.9, 0.7)	0.864
Operative mortality (%)	1.6	0.9	0.7	(−0.2, 1.7)	0.145
Reoperation within 30 days (%)	6.0	4.9	1.1	(−0.9, 3.1)	0.294
Pneumonia (%)	15.4	15.7	−0.3	(−3.4, 2.8)	0.843
Atelectasis (%)	5.2	5.7	−0.5	(−2.5, 1.5)	0.639
Mechanical ventilation for more than 48 h (%)	4.4	3.3	1.1	(−0.6, 2.7)	0.221
Unplanned intubation (%)	4.0	3.3	0.7	(−1.0, 2.4)	0.398
Empyema (%)	1.0	1.2	−0.2	(−1.2, 0.8)	0.687
Deep vein thrombosis (%)	1.0	1.6	−0.6	(−1.8, 0.5)	0.292
Superficial surgical site infection (%)	7.5	7.9	−0.4	(−2.8, 2.1)	0.770
Deep surgical site infection (%)	3.6	3.8	−0.2	(−1.9, 1.6)	0.828
Organ surgical site infection (%)	8.3	7.0	1.3	(−1.0, 3.7)	0.261
Delayed gastric emptying (%)	1.9	1.5	0.3	(−0.9, 1.5)	0.581

*Note:* Adjusted risks, means, and differences were estimated using inverse probability of treatment weighting with G‐computation within the retrosternal subgroup.

Abbreviation: CI, confidence interval.

## 4 Discussion

This study is the first to evaluate the impact of feeding enterostomy creation and enterostomy type on short‐term postoperative outcomes following esophagectomy for esophageal or esophagogastric junction cancer using a large‐scale national database. Owing to the robust sample size and comprehensive coverage of the Japanese NCD [12], these findings provide clinically meaningful and generalizable insights into contemporary practice and the role of feeding enterostomy in MIE. Although enterostomy was historically considered beneficial for perioperative nutritional support, particularly in open esophagectomy, our findings question its routine use in the setting of modern MIE [4].

In this study, feeding enterostomy creation was associated with a higher incidence of several postoperative complications, including reoperation, pneumonia, atelectasis, and prolonged mechanical ventilation. The increased risk of reoperation is likely related to stoma‐associated complications such as obstruction or leakage. Importantly, reoperation due to bowel obstruction was more frequent in the enterostomy group despite a similar overall incidence of bowel obstruction. This finding suggests that bowel obstruction events in the enterostomy group may have been more severe or less responsive to conservative treatment, and it should be recognized as a potential enterostomy‐related morbidity. The higher rate of respiratory complications in the enterostomy group may be partly explained by the greater prevalence of COPD, a known risk factor for postoperative pneumonia and prolonged ventilation [13, 14], which was not included in the propensity score model and may have contributed to residual confounding. Additionally, reconstruction route, the extent of abdominal manipulation, and institutional experience may influence respiratory complications after MIE [15, 16]. Although IPTW was used to balance age, ASA‐PS, laparoscopic approach, reconstruction route, and institutional volume, residual confounding due to comorbidities (including COPD), unmeasured factors, or subtle differences in technique and case selection cannot be excluded. Importantly, despite the increased incidence of certain complications, no differences were observed in short‐term mortality or overall postoperative length of hospital stay.

Conversely, several potential advantages of feeding enterostomy were identified. Delayed gastric emptying and deep vein thrombosis occurred significantly less frequently in the enterostomy group, suggesting that early enteral feeding and improved mobilization contribute to enhanced postoperative recovery. Superficial and deep SSI also tended to be less frequent in the enterostomy group, although these differences did not reach statistical significance. This trend may reflect more stable postoperative nutritional support and earlier rehabilitation, although the underlying mechanism remains speculative.

Previous studies have supported the clinical value of enteral access after MIE. A single‐center randomized controlled trial of thoracoscopic esophagectomy demonstrated that enteral nutrition via jejunostomy reduced early postoperative weight loss and decreased postoperative pneumonia compared with parenteral nutrition [7], highlighting the potential nutritional and respiratory benefits of structured enteral feeding. Conversely, our nationwide analysis indicates that enterostomy creation may be associated with increased postoperative morbidity and complications, underscoring the importance of careful patient selection and meticulous surgical techniques.

In accordance with the 2025 ESPEN guideline on clinical nutrition in surgery, early nutritional support (preferably via the enteral route) should be considered when adequate oral intake is not anticipated after major upper gastrointestinal surgery, particularly in patients at nutritional risk [17]. Collectively, our findings suggest that feeding enterostomy confers physiologic benefits in selected populations, such as patients with significant comorbidities, advanced age, or malnutrition [10]. However, given the potential for procedure‐related morbidity and complications, enterostomy use should be individualized based on patient risk profiles and nutritional needs rather than applied routinely, consistent with ESPEN recommendations [17].

To enable a meaningful comparison between gastrostomy and jejunostomy, the analysis was limited to cases reconstructed via the retrosternal route as gastrostomy was almost exclusively performed in this subset [15]. Within this subgroup, the operative time was significantly shorter in the jejunostomy group, whereas postoperative length of stay was significantly shorter in the gastrostomy group. The shorter total operative time observed in the jejunostomy group may reflect inter‐institutional practice patterns, including differences in concomitant procedures and perioperative workflows, rather than the enterostomy technique itself. Therefore, these findings should not be interpreted as a direct comparison of the time required to create jejunostomy versus gastrostomy. Because bowel obstruction and reoperation for bowel obstruction were rare, adjusted analyses were not performed; descriptively, these events occurred more frequently in the jejunostomy group. No significant differences were observed between the two groups in other postoperative complications. Based on these findings, gastrostomy represents a safer option with respect to mechanical complications, particularly in retrosternal reconstruction [10, 11]. Conversely, in posterior mediastinal reconstruction, where gastrostomy is technically more challenging, jejunostomy remains a practical and commonly used option [11]. Ultimately, the choice of enterostomy type should be guided by surgical feasibility, institutional preference, and patient‐specific risk profiles.

Additionally, technical refinements to prevent stoma‐related mechanical complications are essential. These include creating a tension‐free and straight enterostomy limb without kinking or torsion, ensuring secure fixation to the abdominal wall, and careful placement to avoid compression or angulation during reconstruction as such issues may contribute to reoperation [10, 18].

Several limitations warrant consideration. First, the analysis was limited to short‐term outcomes during the index hospitalization and within 30 days; long‐term nutritional status, bodyweight changes, adjuvant therapy completion, and survival could not be evaluated. Second, although key surgical and institutional variables were balanced using propensity score weighting, residual confounding due to unmeasured factors cannot be excluded. Third, because this study relied on data from the Japanese NCD, misclassification is possible, including incomplete capture of enterostomy creation and the inability to distinguish surgically created enterostomy from nasoenteric feeding, which may have influenced group allocation and outcomes. Finally, prior studies indicate that postoperative enteral feeding may support longer‐term recovery, body weight maintenance, and tolerance to adjuvant therapy [19], but these endpoints were not assessable in the present dataset. Further studies are needed to clarify long‐term outcomes and optimize patient selection and enterostomy techniques.

In summary, feeding enterostomy creation was associated with both potential benefits and increased postoperative morbidity following MIE. These findings suggest that routine or uniform placement may be avoided in contemporary practice. When enteral access is considered necessary, decisions may be individualized based on patient condition and anticipated postoperative nutritional needs, with careful attention to the technique and enterostomy type, and gastrostomy may be preferable to jejunostomy in retrosternal reconstruction.

## 5 Conclusions

Feeding enterostomy creation during MIE was associated with increased postoperative morbidity, including higher reoperation and respiratory complications, although delayed gastric emptying and deep vein thrombosis were less frequent. These findings suggest that routine placement may be avoided and enterostomy may be considered on an individualized basis when reliable enteral access is deemed necessary.

## Author Contributions

**Eisuke Booka:** conceptualization, methodology, investigation, validation, writing – original draft, writing – review and editing, visualization, project administration, formal analysis, data curation. **Shinya Hirakawa:** software, resources, data curation, formal analysis, writing – review and editing, validation, visualization, methodology. **Hisateru Tachimori:** methodology, validation, visualization, writing – review and editing, formal analysis, software, data curation, resources. **Koji Tanaka:** writing – review and editing, conceptualization. **Hideki Ueno:** writing – review and editing. **Yasue Kimura:** writing – review and editing. **Ken Shirabe:** writing – review and editing, supervision. **Hiroya Takeuchi:** writing – review and editing, supervision, writing – original draft.

## Funding

The authors have nothing to report.

## Ethics Statement

Approval of the research protocol: Institutional Review Board of Hamamatsu University School of Medicine. Registry and the registration No. of the study/trial: ID: 23–254.

## Conflicts of Interest

Hideki Ueno, Ken Shirabe, and Hiroya Takeuchi are members of the editorial board of *Annals of Gastroenterological Surgery*. Shinya Hirakawa and Hisateru Tachimori were affiliated with the Endowed Course for Health System Innovation at Keio University, which was funded by Takeda Pharmaceutical Company Limited, until March 31, 2024. They are currently affiliated with the Department of Healthcare Quality Assessment at the University of Tokyo, which receives grants from the National Clinical Database, Johnson & Johnson K.K., Nipro Corporation, and Intuitive Surgical Sàrl. The remaining authors declare no conflicts of interest or sources of funding relevant to this study.

## Data Availability

The data that support the findings of this study are available on reasonable request from the corresponding author. The data are not publicly available due to privacy or ethical restrictions.
